# Persistent COVID-19 Infection in an Immunocompromised Host: A Case Report

**DOI:** 10.7759/cureus.68679

**Published:** 2024-09-04

**Authors:** Rashmita Das, Rajesh P Karyakarte, Suvarna Joshi, Marie Joy, Abhay Sadre

**Affiliations:** 1 Microbiology, Byramjee Jeejeebhoy Government Medical College and Sassoon General Hospitals, Pune, IND; 2 Nephrology, Ruby Hall Clinic, Pune, IND

**Keywords:** covid-19, immunocompromised hosts, persistent rt-pcr positivity, sars-cov-2, viral evolution

## Abstract

This case report highlights the prolonged SARS-CoV-2 reverse transcriptase polymerase chain reaction positivity in a 32-year-old immunocompromised male with a history of kidney transplants and chronic kidney disease. The whole genome sequencing of nasopharyngeal samples for SARS-CoV-2 collected 12 days apart showed the presence of the BA.1.1 Omicron variant. It revealed evidence of intra-host viral evolution, showing the development and loss of specific mutations over time. This report emphasizes the need for continuous monitoring strategies for immunocompromised patients, as they may serve as reservoirs for viral evolution and potentially give rise to immune escape variants.

## Introduction

SARS-CoV-2, the virus responsible for COVID-19, has exhibited significant genetic variability, leading to the emergence of multiple variants with different viral properties [1]. The World Health Organization (WHO) evaluates the effects of highly divergent SARS-CoV-2 variants on global public health by assessing their transmissibility, clinical presentation, disease severity, impact on diagnostics, and therapeutics. Based on this assessment, the WHO designates and updates the classifications for variants of concern, variants of interest, or variants under monitoring [2]. The continued evolution and spread of these variants underline the virus's capacity for adaptation, which can complicate control and prevention efforts [1].

Several hypotheses have been proposed explaining the origin of highly divergent SARS-CoV-2 variants. These include undetected circulation in regions with limited genomic surveillance, zoonotic transmission from animal reservoirs, and emergence in chronically infected individuals [3-5]. Though proving any of these hypotheses is challenging, the hypothesis of variants emerging in chronically infected individuals has gained increasing interest in recent times [5]. In most infected individuals, the illness lasts for one to two weeks [1]. It takes an average of 17.5 days to transition from a positive to a negative test, with 90% of patients testing negative within 28 days [6]. On the other hand, chronic SARS-CoV-2 infections are characterized by evidence of active viral replication for more than 21 days, often lasting months or even over a year. These prolonged infections have been observed primarily in immunocompromised individuals, including those with hematologic cancers, AIDS, organ transplants, or autoimmune disorders [5]. Such individuals may exhibit variations in the degree of viral shedding, immune clearance, and disease severity [1]. Currently, the knowledge of the dynamics of these infections and viral evolution is largely based on individual case reports [5].

Here, we report an immunocompromised patient who remained reverse transcriptase polymerase chain reaction (RT-PCR) test positive for SARS-CoV-2 for more than a year from initial infection. Notably, genomic analysis of positive nasopharyngeal samples collected at two different time points revealed the emergence of an additional mutation in the genome.

## Case presentation

In May 2021, a 32-year-old male with a history of chronic kidney disease of unknown etiology initially presented to the hospital with a history of fever for five days. He had a significant medical history that included a kidney transplant eight years earlier. After the first transplant, the patient encountered complications with antibody-mediated rejection of the transplanted kidney, which was treated with splenic irradiation, double filtration, and administration of alemtuzumab and everolimus. Subsequently, he underwent a deceased donor kidney transplant and has been on a regimen of immunosuppressives: everolimus, mycophenolate mofetil, and prednisolone. His medical journey was further complicated by renal tuberculosis, for which he was treated with anti-tubercular therapy for a year. He had also received two doses of the COVID-19 vaccine (Covaxin®; Bharat Biotech).

**Figure 1:**
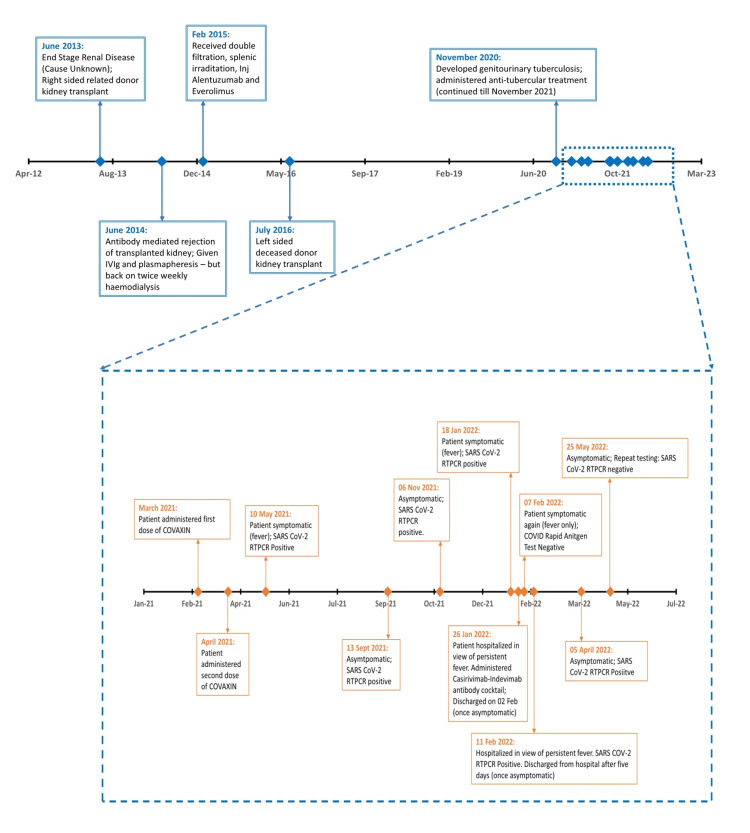
Timeline of the events for the immunocompromised individual SARS‑CoV‑2: severe acute respiratory syndrome coronavirus 2, COVID: coronavirus disease, RT-PCR: reverse transcriptase polymerase chain reaction Image Credit: Dr. Marie Joy

During the first episode of fever in May 2021, the patient tested positive for SARS-CoV-2 (RT-PCR positive for SARS-CoV-2) but exhibited only mild symptoms, for which he was managed by home isolation. Over the following months (as illustrated in Figure 1), he consistently tested positive by RT-PCR despite remaining asymptomatic. In January 2022, he still tested positive for SARS-CoV-2 and developed symptoms again, including fever and a mild cough. As the fever persisted for over a week, he was admitted to the hospital. On admission, he received treatment with a monoclonal antibody cocktail (casirivimab-imdevimab) and remdesivir, with full recovery of symptoms within a week of admission.

A week after discharge, he experienced similar symptoms again. However, the rapid antigen test (RAT) for COVID-19 was negative. Despite this, he remained febrile and was re-hospitalized, where RT-PCR testing confirmed a positive result. Persistent positivity was noted in subsequent SARS-CoV-2 RT-PCR tests over the next months, until he finally tested negative in May 2022, marking over a year since his initial positive test. Figure 1 illustrates the entire timeline of events for the immunocompromised patient.

**Figure 2:**
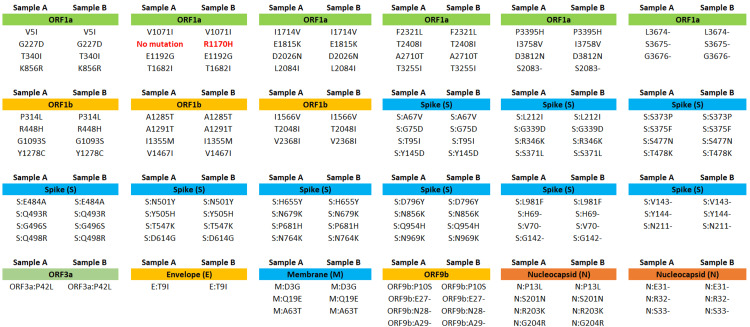
Amino acid changes identified in both samples sequenced ORF: open reading frame Image Credit: Dr. Rashmita Das

**Figure 3:**
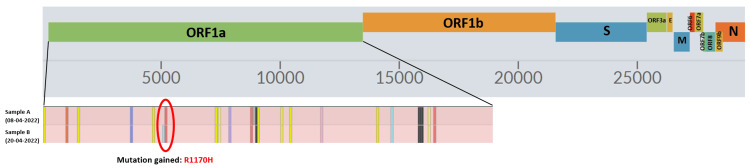
Additional amino acid change R1170H in the ORF 1a ORF: open reading frame Image Credit: Dr. Rashmita Das

Despite testing positive for SARS-CoV-2 multiple times, only two nasopharyngeal swab samples were available for genomic analysis. To study the potential intra-host evolution of the virus, whole genome sequencing was performed on these two samples, collected 12 days apart on April 8, 2022, and April 20, 2022. Both samples were identified as the BA.1.1 Omicron variant, with a coverage of 97.9% (April 8, 2024) and 98.4% (April 20, 2024). All amino acid changes identified in the samples are shown in Figure 2. The second sample showed an additional amino acid change, R1170H (nucleotide change G3774A), in the open reading frame 1a (ORF 1a) (Figure 3).

## Discussion

Persistent SARS-CoV-2 RT-PCR positivity can be attributed to a combination of viral and host factors. Viral factors include viral load dynamics and genomic variations, contributing to prolonged shedding and delayed clearance, thus leading to persistently positive RT-PCR results [7]. Host factors such as age, comorbidities, and immune response play critical roles in viral clearance [8]. An important host factor responsible for prolonged viral shedding is immunosuppression. Immunocompromised individuals represent a vulnerable population with a diminished ability to fight infections. This population is less protected by vaccines and may not develop sufficient immunity to clear the virus, thus leading to prolonged viral shedding and delayed viral clearance. Several case reports and case series have documented prolonged infections, lasting hundreds of days, in immunocompromised individuals [1,9-11]. They may potentially serve as reservoirs for viral replication in the background of partial immune control. Therefore, they may facilitate rapid multistage viral evolution (saltation) and pose public health risks due to the emergence of immune escape variants [12,13]. However, this is in contrast with a slower rate of adaptive evolution observed during the transmission chain of acute infections [5].

It is important to highlight that in the present study, the patient received a monoclonal antibody cocktail (casirivimab-imdevimab). Studies have shown that in patients treated with these monoclonal antibodies, mutations can occur within the antibody epitopes in the spike (S) protein. These mutations can reduce or completely negate the antibody affinity and neutralizing activity, indicating that they are driven by immune evasion [14,15].

A significant proportion of these mutational changes occur in the spike (S) protein. In spite of being only 13% of the proteome, mutational changes in this protein are consistent with adaptive evolution [13]. Supporting the potential biological significance of immune-escape mutations, spike mutations like Y453F, E484K, and del69/70 potentially influence the affinity of the SARS-CoV-2 virus for ACE-2 receptors, evading the immune response and enhancing the efficiency of viral entry, respectively [16]. In the present case, an additional mutation at the 1170th amino acid position (R1170H) in the ORF 1a protein, corresponding to a mutation in non-structural protein 2 (nsp-2) [17], was identified. The nsp-2, an endosome-associated protein, interacts with a variety of host proteins and takes part in several biological processes, including viral replication, host immune regulation, mitochondrial biogenesis, and endosomal transport. Although mutations in nsp-2, such as T85I in the B.1.526/B.1.427/B.1.429 variants of SARS-CoV-2, have been linked to increased transmissibility and pathogenicity [18], the impact of the R1170H mutation on the viral characteristics remains unclear. Despite this uncertainty, the emergence of the R1170H mutation over time in the case study serves as evidence of ongoing intra-host viral evolution in an immunocompromised patient. Unfortunately, genomic analysis of all nasopharyngeal samples collected from the onset of infection in the case study was not possible. Such an analysis would have provided deeper insights into the intra-host viral evolution in this scenario.

## Conclusions

This case report highlights the critical role of immunocompromised individuals in the evolution of SARS-CoV-2. The described case details prolonged viral shedding in such patients that can facilitate significant genetic changes in the viral genome, potentially leading to the emergence of immune escape variants. It underscores the urgent need for enhanced monitoring and understanding of viral dynamics in immunocompromised hosts, as they may serve as reservoirs for viral evolution. Given the potential for these variants to exhibit increased transmissibility and pose a public health threat, it is essential to prioritize genomic surveillance for this vulnerable population to mitigate the risk of future outbreaks. Furthermore, ongoing studies of similar cases will likely provide valuable insights into the pathogenesis, evolution, and immune response to SARS-CoV-2 in immunocompromised individuals.
